# Prognostic Utility of Arterial Spin Labeling in Traumatic Brain Injury: From Pathophysiology to Precision Imaging

**DOI:** 10.3390/neurosci6030073

**Published:** 2025-08-04

**Authors:** Silvia De Rosa, Flavia Carton, Alessandro Grecucci, Paola Feraco

**Affiliations:** 1 Centre for Medical Sciences—CISMed, University of Trento, Via S. Maria Maddalena 1, 38122 Trento, Italy; silvia.derosa@unitn.it (S.D.R.); flavia.carton@unitn.it (F.C.); alessandro.grecucci@unitn.it (A.G.); 2 Department of Psychology and Cognitive Sciences of Trento, University of Trento, 38068 Rovereto, Italy

**Keywords:** arterial spin labeling (ASL), traumatic brain injury (TBI), cerebral blood flow (CBF), MRI, perfusion imaging, arterial transit time (ATT)

## Abstract

Background: Traumatic brain injury (TBI) remains a significant contributor to global mortality and long-term neurological disability. Accurate prognostic biomarkers are crucial for enhancing prognostic accuracy and guiding personalized clinical management. Objective: This review assesses the prognostic value of arterial spin labeling (ASL), a non-invasive MRI technique, in adult and pediatric TBI, with a focus on quantitative cerebral blood flow (CBF) and arterial transit time (ATT) measures. A comprehensive literature search was conducted across PubMed, Embase, Scopus, and IEEE databases, including observational studies and clinical trials that applied ASL techniques (pCASL, PASL, VSASL, multi-PLD) in TBI patients with functional or cognitive outcomes, with outcome assessments conducted at least 3 months post-injury. Results: ASL-derived CBF and ATT parameters demonstrate potential as prognostic indicators across both acute and chronic stages of TBI. Hypoperfusion patterns correlate with worse neurocognitive outcomes, while region-specific perfusion alterations are associated with affective symptoms. Multi-delay and velocity-selective ASL sequences enhance diagnostic sensitivity in TBI with heterogeneous perfusion dynamics. Compared to conventional perfusion imaging, ASL provides absolute quantification without contrast agents, making it suitable for repeated monitoring in vulnerable populations. ASL emerges as a promising prognostic biomarker for clinical use in TBI. Conclusion: Integrating ASL into multiparametric models may improve risk stratification and guide individualized therapeutic strategies.

## 1. Introduction

Traumatic brain injury (TBI) is a major global cause of death and disability, affecting individuals across all age groups and imposing a substantial burden on healthcare systems and societal productivity [1]. In both Europe and the United States, TBI results in several hundred emergency visits and hospitalizations per 100,000 people annually, with older adults being especially vulnerable to poor outcomes [2]. Despite improvements in acute management and neurocritical care [3], accurately predicting long-term functional recovery in TBI remains a significant challenge.

Neuroimaging plays a pivotal role in the initial assessment and follow-up of TBI [4]. However, conventional computerized tomography (CT) and magnetic resonance imaging (MRI) lack sensitivity to detect microstructural and functional abnormalities that underlie persistent cognitive and behavioral impairments. In this context, the search for reliable imaging biomarkers that can support early prognosis and personalized intervention has gained momentum [5].

Arterial Spin Labeling (ASL) is an advanced non-invasive MRI technique that quantifies cerebral blood flow (CBF) by using arterial water as an endogenous tracer [6]. ASL works by inverting the magnetization of arterial blood water upstream from the brain region of interest, followed by a post-labeling delay (PLD) that allows the labeled bolus to reach the cerebral tissue [7]. The resulting signal intensity allows quantitative estimation of CBF in units of mL/100 g/min. Recent studies have investigated its application in TBI, reporting promising findings across a range of severity levels and time points [8].

This review examines the prognostic utility of ASL in TBI, highlighting its potential in monitoring cerebral blood flow and predicting clinical outcomes. We discuss its physical principles, technical variants, and clinical applications, and evaluate how ASL-derived perfusion metrics relate to functional and cognitive outcomes. Furthermore, we explore how the ASL can complement existing clinical models and its integration into machine learning frameworks for outcome prediction.

## 2. Epidemiology, Socioeconomic Impact, and the Need for Biomarkers in TBI

According to data from the Centers for Disease Control and Prevention (CDC), the United States records approximately 403 emergency department visits and 85 hospital admissions for TBI per 100,000 people annually, underscoring the significant public health and economic impact of TBI [9]. A similar situation is observed in Europe, where the total cost of TBI was estimated at €33 billion in 2010, equivalent to approximately 49.7 billion USD in 2017, with 41% of this figure attributed to direct healthcare expenses and 59% to indirect costs, such as loss of productivity [10]. These figures highlight the urgent need to implement effective prevention and clinical management strategies that consider not only clinical outcomes but also the economic sustainability of healthcare systems. Despite age-related differences, all population groups affected by TBI exhibit high rates of mortality and disability [11]. Older adults generally experience significantly worse clinical outcomes compared to their younger counterparts [11]. Nonetheless, admission to specialized trauma centers has contributed to a notable reduction in post-injury mortality, supported by recent advances in intensive care and primary prevention strategies [12]. Current diagnostic approaches to TBI primarily rely on clinical evaluation and neuroimaging modalities such as CT and MRI. However, these tools often fail to detect microscopic damage and are limited in their ability to predict functional outcomes. In this context, blood-based biomarkers are emerging as valuable complementary tools. Several candidates, such as GFAP, UCH-L1, S100B, NF-L, and tau, have shown promising clinical potential in early diagnosis, risk stratification, outcome prediction, and treatment monitoring [13]. A summary of their principal characteristics and clinical applications is provided in Table 1. Although further systematic studies and large-scale validations are needed, integrating these biomarkers into clinical models holds promise for improving diagnostic accuracy, reducing costs, and minimizing unnecessary radiation exposure.

## 3. Pathophysiology of Cerebral Blood Flow After Traumatic Brain Injury

**Figure 1:**
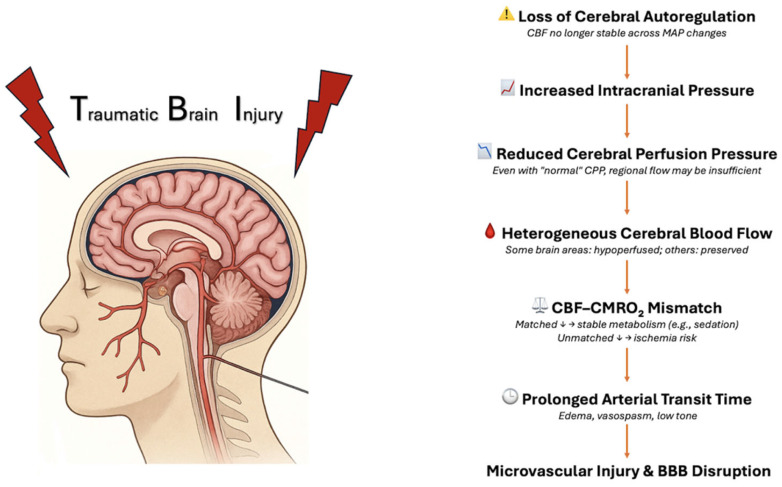
Mechanisms of Cerebral Perfusion Impairment Following TBI.

TBI profoundly and significantly alters cerebral hemodynamics. After such an injury, the standard mechanisms that help keep CBF stable can become compromised (Figure 1) [14].

## 4. Injury

In the healthy brain, cerebral autoregulation allows CBF to remain relatively stable even if blood pressure changes. However, in many patients with TBI, especially those with diffuse brain damage, this autoregulatory mechanism becomes disrupted [15]. As a result, cerebral perfusion pressure (CPP) becomes the primary factor driving CBF, meaning that even if CPP values appear normal (for example, between 50 and 70 mmHg), blood flow to some brain regions might still be insufficient [8]. Increased intracranial pressure (ICP), which often occurs after trauma, further lowers CPP, making the situation worse [8]. Several studies using imaging techniques like PET or xenon-enhanced CT have shown that CBF after TBI is not uniform; some areas receive enough blood, while others do not [16]. However, reduced CBF does not necessarily indicate cerebral ischemia [17]. Whether a brain region suffers from a lack of oxygen depends on the balance between CBF and the cerebral metabolic rate of oxygen (CMRO_2_) [18]. When both CBF and CMRO2 decrease simultaneously, as seen in states of deep sedation, tissue viability may be preserved despite reduced perfusion. In contrast, if blood flow drops more than the brain’s oxygen use, cellular energy failure and apoptosis may ensue [18].

TBI also alters the temporal dynamic of blood flow to reach brain tissue, a parameter known as arterial transit time (ATT) [7]. Factors such as edema, vasospasm, and poor vascular tone can lead to prolonged ATT, making oxygen delivery less efficient. Simultaneously, damage to the small blood vessels (microcirculation) caused by inflammation, clot formation, or swelling can further restrict the flow of oxygen to brain cells, even when CBF seems adequate [19]. Finally, the disruption of the blood–brain barrier, together with the release of toxic substances like glutamate and inflammatory cytokines, worsens swelling and causes further injury [20]. Collectively, these macrovascular and microvascular alterations create a fragile state where even small drops in perfusion or pressure can cause serious harm. Accordingly, the use of monitoring CBF through tools like transcranial Doppler, brain oxygen sensors, and multimodal neuromonitoring has become increasingly important in managing patients with severe TBI [21].

## 5. Methods

This narrative review was conducted following a structured PICO framework to explore the prognostic role of ASL in TBI. We included original observational studies and clinical trials that enrolled at least 10 adult or pediatric patients with TBI of any severity, who underwent quantitative ASL MRI (e.g., pCASL, PASL, VSASL, multi-PLD, Hadamard encoding) in the acute or subacute phase. A comprehensive literature search was performed across PubMed, Embase, Scopus, and IEEE Xplore databases. The search strategy included combinations of terms such as “arterial spin labeling,” “ASL,” “traumatic brain injury,” “TBI,” “prognosis,” “cerebral blood flow,” and “arterial transit time.” Only English-language articles with full text available were considered. Studies without human data, with fewer than 10 patients, or lacking prognostic outcomes were excluded.

## 6. ASL Techniques and Quantitative Parameters

**Figure 2:**
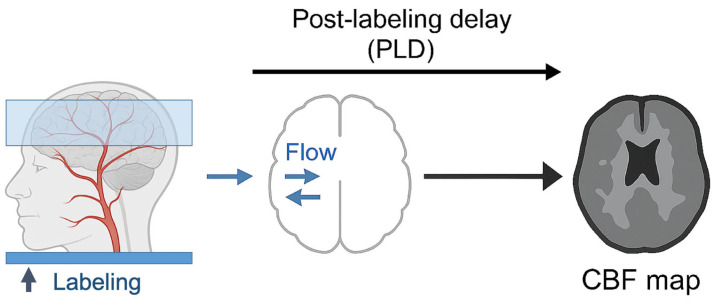
Schematic illustration of arterial spin labeling (ASL) imaging principles.

In ASL, arterial blood water serves as an endogenous tracer using magnetic labeling. This process begins with the application of selective radiofrequency (RF) pulses to the cervical arteries, below the skull base, which invert the longitudinal magnetization of inflowing blood. After a post-labeling delay (PLD) that allows the tagged blood to reach the cerebral vasculature, image acquisition is performed. The subtraction of the control (non-labeled) image from the labeled image yields a perfusion-weighted map that reflects CBF (Figure 2).

This diagram depicts the ASL acquisition workflow. Magnetically labeled arterial blood is generated by applying a radiofrequency (RF) pulse to a plane located at the base of the skull, in the feeding arteries (labeling region). After a defined PLD, the labeled blood flows into the imaging volume, where it exchanges with tissue water, altering the MRI signal. Subtraction of control (non-labeled) from label images yields perfusion-weighted maps that reflect regional CBF. 

ASL comprises multiple implementation strategies that vary by the method of labeling arterial blood, the delay before image acquisition, and the post-processing approach [22,23,24,25]. The most widely accepted classification includes pseudocontinuous ASL (pCASL), pulsed ASL (PASL), velocity-selective ASL (VSASL), and time-encoded or multi-delay ASL protocols.

Principal characteristics of each ASL technique are summarized in Table 2.

pCASL uses a train of RF pulses and gradient fields to mimic continuous labeling, offering high labeling efficiency and superior SNR. It is currently the recommended standard for clinical implementation by the ISMRM–ESMRMB consensus [23]. Quantification in pCASL is typically based on a single-compartment model, with assumptions about T1 of blood, labeling efficiency (α), and post-labeling delay (PLD).

PASL involves the instantaneous inversion of a thick slab proximal to the imaging region. Techniques such as FAIR (Flow-sensitive Alternating Inversion Recovery), QUIPSS II (Quantitative Imaging of Perfusion using a Single Subtraction), and PICORE (Proximal Inversion with Control for Off-Resonance Effects) fall under this category. PASL offers faster acquisitions but suffers from lower SNR and increased sensitivity to ATT variability.

VSASL departs from spatial labeling and instead tags blood based on flow velocity thresholds. It is less sensitive to ATT variability and particularly suited for populations with unpredictable or prolonged transit times, such as severe TBI or cerebrovascular compromise. However, it is technically demanding and not yet widely implemented commercially.

Multi-PLD and Time-Encoded ASL (e.g., Hadamard-encoded pCASL) enable the acquisition of perfusion-weighted images at multiple timepoints after labeling. This allows the simultaneous estimation of CBF, ATT, and bolus arrival profiles, yielding a richer hemodynamic profile and mitigating the confounding effects of variable ATT.

Quantitative modeling in ASL typically follows the Buxton model [26], with key parameters including the following:

- Labeling duration (τ): commonly 1.5–2.0 s for pCASL;
- Post-labeling delay (PLD): typically, 1.5–2.0 s in adults, adjusted in pediatric or vascular-compromised populations;
- T1 of blood (T1b): ~1650 ms at 3T;
- Labeling efficiency (α): 0.85–0.95 for pCASL, lower in PASL;
- M0 reference image: used to normalize signal to absolute CBF (ml/100 g/min).

Post-processing steps typically involve absolute CBF quantification using an M0 reference, generation of ATT and bolus dispersion maps, atlas registration, normalization, and smoothing. Open-source pipelines like BASIL/FSL and ASLPrep provide reproducible and standardized processing workflows, facilitating consistency [27,28].

Unlike other MRI perfusion methods such as dynamic susceptibility contrast (DSC-MRI) and dynamic contrast-enhanced imaging (DCE-MRI), ASL offers the unique advantage of being non-contrast-enhanced, repeatable, and applicable even in pediatric and renal-impaired populations [29,30]. While DSC-MRI provides higher spatial and temporal resolution and has greater sensitivity in acute stroke and tumor imaging, it relies on gadolinium-based contrast agents. It is limited to patients with renal insufficiency. DCE-MRI, primarily used to assess permeability and perfusion in tumors, also involves contrast administration and more complex modeling assumptions.

In contrast, ASL provides absolute quantification of CBF and is well-suited for longitudinal studies and clinical follow-up without the risks associated with exogenous tracers. It is particularly advantageous for conditions with diffuse or subtle perfusion abnormalities, such as TBI, neurodegenerative diseases, and psychiatric disorders. ASL has already shown broad clinical utility in the study of dementias (Alzheimer’s, frontotemporal), epilepsy, assessment of cerebrovascular reserve (CVR), hypoxic-ischemic encephalopathies, psychiatric disorders, and brain oncology (grading and treatment response) [21,22,23,24,25,26,27,28,29,30,31,32,33]. Recent advancements in high-resolution ASL sequences, including 3D-GRASE, spiral acquisition, and simultaneous multi-slice (SMS) readouts, combined with quality-controlled analysis pipelines, have significantly improved the precision and reproducibility of CBF measurements. These innovations support the integration of ASL into precision medicine frameworks and enhance its role as a biomarker in prognostic stratification [28,33].

## 7. Technical Limitations and Artifacts

Despite the numerous strengths of ASL, its clinical application, especially in the context of TBI, is limited by a series of well-recognized technical challenges and artifacts that affect image reliability and interpretation. A first major limitation is motor sensitivity. Since ASL techniques often have a relatively low signal-to-noise ratio (SNR), especially in variants such as PASL, the acquisition is particularly vulnerable to patient movement. This is especially problematic in vulnerable populations such as children, elderly patients, or those in intensive care settings, where compliance is often poor and motion correction is complex to implement reliably [29,34]. Susceptibility artifacts and partial volume effects pose another important challenge. Indeed, magnetic field inhomogeneities near bone-air interfaces and within contusional or hemorrhagic lesions may cause spatial distortion and signal loss. This limit could be addressed by the integration of CT scan with ASL, which could assist in correlating structural findings with perfusion abnormalities, enhancing diagnostic confidence in TBI evaluation. Indeed, as highlighted by Stein et al. [35], CT remains a rapid and cost-effective modality for detecting structural lesions in TBI, and its use alongside ASL can help distinguish true hypoperfusion from artifacts related to hemorrhages, calcifications, or bone-air interfaces that may affect ASL quality. This multimodal CT-ASL approach can support precision imaging and reduce misinterpretation due to artifacts, aligning with practical clinical workflows in TBI management.

Additionally, perfusion values can be underestimated or confounded in areas of tissue heterogeneity due to partial volume averaging [30]. In most clinical settings, ASL is implemented with a single PLD, assuming a uniform ATT. However, this assumption often fails in TBI, where autoregulatory mechanisms are compromised and transit times may be delayed or heterogeneous across regions. This can lead to a substantial underestimation of CBF, particularly in areas with impaired perfusion [23,34].

To overcome this problem, the integration of MRI-based perfusion imaging with neurophysiological studies, such as EEG or evoked potentials, may help to overcome artifacts and improve the interpretation of ASL findings in TBI. Neurophysiological monitoring can provide functional information that aligns with ASL-derived perfusion maps, enabling clinicians to distinguish true hypoperfusion from artifact-related changes and to identify functionally impaired brain regions requiring targeted management. This combined MRI–neurophysiological approach can strengthen diagnostic confidence and support precision monitoring in TBI care.

Moreover, the problem of inter-vendor variability is also relevant, as different MRI manufacturers (e.g., Siemens, GE, Philips) implement ASL using proprietary labeling strategies, default parameters, and reconstruction algorithms. These discrepancies affect labeling efficiency, PLD values, and background suppression, making cross-site reproducibility difficult and limiting the comparability of quantitative CBF data across centers [28].

Finally, the absence of standardized perfusion phantoms represents a critical bottleneck for multicenter trials. While phantoms exist for structural or diffusion imaging, there is no universally accepted reference standard for ASL perfusion. This complicates calibration and quality assurance procedures and restricts the possibility of harmonized acquisition and analysis pipelines in clinical research [33].

## 8. Prognostic Value of Arterial Spin Labeling in Traumatic Brain Injury

One of the earliest studies highlighting the prognostic value of cerebral perfusion in severe traumatic brain injury was conducted by Kellly et al. [36], using xenon-133 imaging. They found that episodes of cerebral hyperemia (CBF > 55 mL/100 g/min) coupled with elevated intracranial pressure (>20 mmHg) were associated with poor clinical outcomes. In contrast, hyperemia in the absence of intracranial hypertension was more frequently associated with a favorable prognosis [36]. These findings highlight the role of CBF monitoring as an early prognostic indicator and suggest the presence of distinct pathophysiological mechanisms underlying post-traumatic perfusion patterns [36]. The advent of ASL has made cerebral perfusion measurement more accessible in TBI research. Cross-sectional studies have confirmed the association between cerebral hypoperfusion and functional outcomes. For instance, Ware et al. [37] demonstrated that reduced cortical CBF at 3 months post-injury was correlated with poorer cognitive and functional recovery up to one year. Similarly, Ding et al. reported persistent impairment in dynamic cerebral autoregulation (dCA) among chronic symptomatic TBI patients, with lower dCA values predicting worse neuropsychological performance, suggesting a link between microvascular dysfunction and long-term deficits [38]. At the regional level, Thomas et al. showed a negative correlation between CBF in limbic regions (specifically, the hippocampus and rostral anterior cingulate cortex) and affective symptoms such as anxiety, depression, and fatigue [39]. These findings underscore the relevance of CBF as a potential biomarker not only for cognitive recovery but also for neuropsychiatric outcomes in chronic TBI. On the technological front, recent studies in acute ischemic stroke have demonstrated the added prognostic value of multi-PLD ASL. For example, Wang et al. integrated radiomic features from multi-delay ASL CBF maps with clinical data to develop machine learning models predicting 90-day outcomes, achieving an AUC of 0.876 [40]. Although focused on stroke, this modeling approach is highly applicable to TBI, where contrast-free imaging is often preferable in critically ill patients, and perfusion heterogeneity may provide insight into outcome stratification. Finally, among the most promising experimental applications, Velocity-Selective ASL (VSASL) stands out. Unlike traditional spatial ASL methods, VSASL labels blood based on its velocity, making it less dependent on ATT and more sensitive to capillary-level flow [40]. This makes it particularly suitable for severe TBI patients in intensive care, where delayed flow and autoregulatory failure may impair standard ASL quantification. As reviewed by Qin et al., VSASL shows strong potential for accurate perfusion mapping in cases of compromised cerebral hemodynamics and may be especially valuable for non-invasive monitoring in neurocritical care settings [41,42]. Additional evidence of progressive CBF decline and persistent hypoperfusion in moderate-to-severe and chronic TBI has been reported by Gaggi et al. [43]; global grey matter CBF reduction has been correlated to cognitive deficits, while in mild TBI, ASL studies suggest both hypo- and hyperperfusion patterns. Technical comparisons also support the superiority of pCASL over PASL in TBI imaging.

## 9. Integrative Prognostic Models in TBI and Future Directions

The integration of ASL perfusion imaging into established prognostic frameworks for TBI represents a promising avenue for improving risk stratification and early outcome prediction, such as the International Mission for Prognosis and Clinical Trials in Traumatic Brain Injury (IMPACT) and the Corticosteroid Randomization after Significant Head Injury (CRASH) model [44,45,46]. ASL is particularly well suited to TBI applications due to its repeatability, absence of exogenous contrast agents, and sensitivity to perfusion alterations that may precede structural changes. However, variability in acquisition protocols remains a key limitation [47]. In prognostic applications, ASL parameters are increasingly being integrated with clinical and imaging predictors using machine learning (ML) techniques. ML algorithms such as random forests, XGBoost, and deep neural networks are well-suited to modeling non-linear interactions in high-dimensional data [48].

These models can incorporate diverse inputs, including GCS scores, pupillary response, CT findings, and regional CBF/ATT maps, to generate individualized outcome predictions. Standard evaluation metrics include area under the ROC curve (AUC) for discrimination, calibration plots for agreement between predicted and observed risk, and decision curve analysis for clinical utility assessment [49].

**Figure 3:**
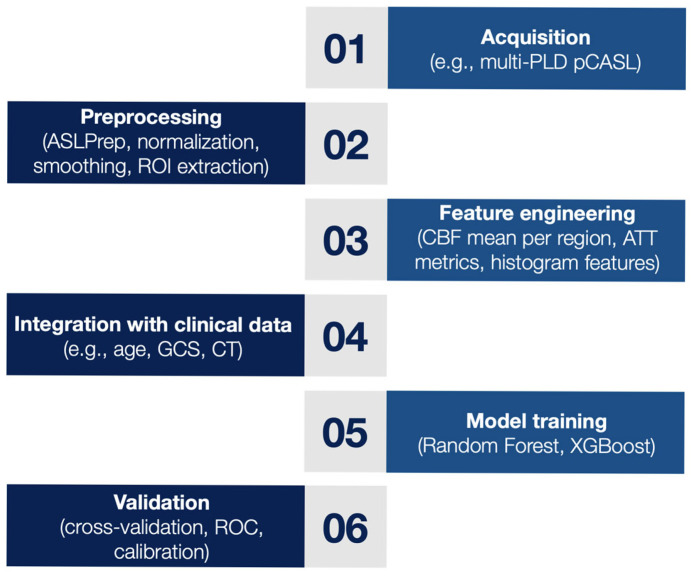
Workflow for Building Prognostic Models Integrating ASL Imaging and Clinical Data in TBI.

Figure 3 illustrates a prototypical workflow for building integrative prognostic models using ASL-derived perfusion metrics and ML techniques in TBI. This approach includes ASL acquisition and preprocessing, feature engineering, integration with clinical data, and model development and validation.

Despite this potential, the development of robust ML-based prognostic tools remains hindered by a lack of large, standardized datasets that include both ASL imaging and long-term clinical outcomes. While consortia like TRACK-TBI and CENTER-TBI provide comprehensive data, ASL sequences are not consistently acquired [50]. Moreover, heterogeneity in ASL protocols introduces noise and limits the generalizability of trained models. Harmonization efforts are essential, including the use of consensus acquisition guidelines, centralized repositories of annotated ASL datasets, and validated processing pipelines to support cross-site reproducibility.

Furthermore, future research should consider the integration of ASL with other techniques, such as DTI, to enhance prognostic accuracy and improve pathophysiological understanding in traumatic brain injury. This multimodal approach would allow the simultaneous assessment of cerebral perfusion and microstructural white matter integrity, providing a more comprehensive evaluation of neurocognitive and neurobehavioral sequelae in TBI [51].

Lastly, recent developments in portable RF sensing systems for CBF monitoring, such as the work by Anwar et al. [52], provide new opportunities for continuous, noninvasive bedside assessment of global CBF trends in TBI. While ASL offers quantitative and spatially resolved perfusion imaging, portable RF systems enable real-time monitoring without the need for MRI facilities, which is particularly advantageous in neurocritical care and resource-limited settings. The integration of RF-based continuous monitoring with ASL-based regional perfusion quantification could enhance precision monitoring in TBI by allowing early detection of secondary insults while providing detailed spatial perfusion maps to guide individualized therapeutic strategies.

The clinical translation of these methods will require not only algorithmic accuracy but also workflow feasibility, regulatory validation, and integration into routine neurocritical care pathways.

## 10. Conclusions

ASL is a promising, non-invasive MRI technique for quantifying cerebral blood flow in patients with TBI, without the need for contrast agents. By detecting perfusion abnormalities linked to cognitive and neuropsychiatric outcomes, ASL provides clinically meaningful insights, particularly when using advanced sequences such as multi-PLD or velocity-selective ASL. This makes it a valuable tool for both acute and chronic phases of TBI management. Compared to conventional perfusion imaging, ASL offers the advantages of repeatability, safety, and applicability in vulnerable populations, including pediatric and renal-impaired patients. Although technical challenges related to motion artifacts remain, transit time variability, and inter-scanner heterogeneity, recent developments in acquisition protocols and post-processing pipelines have significantly improved the reliability of the method. When integrated into multimodal prognostic frameworks, particularly those leveraging ML, ASL-derived features could enhance risk stratification and inform personalized therapeutic decisions. Future directions include the standardization of acquisition protocols across centers, the establishment of shared ASL repositories, and the incorporation of ASL into prospective multicenter studies aimed at clinical translation.

## Figures and Tables

**Table 1:** Diagnostic and Prognostic Roles of Selected Blood Biomarkers in TBI.

Biomarker	Primary Utility	Notes
GFAP (Glial Fibrillary Acidic Protein)	Detection of brain lesions, even in CT-negative patients	Useful in early diagnosis and triage
S100B	Reduces unnecessary CT imaging in mild TBI	High sensitivity; recommended in Scandinavian guidelines
NF-L (Neurofilament Light)	Monitoring of axonal injury	Elevated in both acute and chronic phases of TBI
Tau	Indicator of neurodegenerative processes	Linked to chronic outcomes and repetitive mild TBI
GFAP + UCH-L1	FDA-approved combination for identifying intracranial lesions in mild TBI	Superior diagnostic accuracy compared to single markers

**Table 2:** Technical characteristics, vendor support, advantages, and limitations of different ASL variants applied in TBI.

ASL Technique	Labeling Method	Typical PLD	Vendors	Advantages	Disadvantages	TBI Application
pCASL	Pseudocontinuous arterial spin labeling	1800–2000 ms	Siemens, GE, Philips, Canon, United Imaging	Clinical standard; widely available; absolute CBF quantification	Sensitive to ATT variability; requires accurate hardware calibration	Widely used in clinical TBI for CBF quantification
PASL	Pulsed ASL (FAIR/QUIPSS II/PICORE)	800–1500 ms	Siemens, GE	Short scan times; simple to implement; useful in pediatrics and subacute settings	Low SNR; sensitive to motion and transit time artifacts	Used in pediatric and subacute TBI studies
VSASL	Velocity-selective labeling (velocity-dependent)	N/A	Research-only; limited commercial availability	Insensitive to ATT; useful in low/variable flow (e.g., severe TBI)	Lower spatial resolution; higher noise sensitivity; experimental	Best for acute/severe TBI with delayed or variable blood flow
Multi-PLD ASL	pCASL with multiple post-labeling delays	200–2500 ms	Siemens, Philips, GE (research platforms)	Enables simultaneous CBF and ATT estimation; accurate in heterogeneous perfusion	Longer acquisition time; complex post-processing	Ideal for chronic TBI with heterogeneous perfusion profiles
Dual-echo ASL	Combined ASL and BOLD acquisition	Variable	GE, Siemens, Philips (experimental)	Simultaneous acquisition of perfusion and functional BOLD signal	Experimental; lower perfusion specificity	Applied in cognitive/task-based studies in chronic TBI

This table summarizes key ASL techniques, including pCASL, PASL, VSASL, multi-PLD ASL, and dual-echo ASL, highlighting their labeling schemes, typical post-labeling delays (PLD), signal-to-noise ratio (SNR), supported MRI vendors, and specific advantages and disadvantages. Applications in TBI are also indicated. pCASL is the current clinical standard widely supported across vendors. At the same time, VSASL and dual-echo ASL remain experimental but promising in cases of delayed transit time or for task-based studies. Multi-PLD acquisitions enhance precision in heterogeneous perfusion conditions often observed in chronic TBI.

## Data Availability

Data are contained within the article.
